# Long non-coding RNA BBOX1 antisense RNA 1 increases the apoptosis of granulosa cells in premature ovarian failure by sponging miR-146b

**DOI:** 10.1080/21655979.2022.2031675

**Published:** 2022-02-21

**Authors:** Yuexin Yu, Qian Zhang, Kaixuan Sun, Yinling Xiu, Xiliang Wang, Kaiyue Wang, Li Yan

**Affiliations:** Department of Reproductive Medicine, General Hospital of Northern Theater Command, Shenyang, Liaoning province, PR. China

**Keywords:** BBOX1-AS1, miR-146b, granulosa cell, premature ovarian failure

## Abstract

Long non-coding RNA (lncRNA) BBOX1 antisense RNA 1 (BBOX1-AS1) was reported to participate in ovarian cancer, while its role in other ovarian disorders is unclear. We speculated that BBOX1-AS1 could interact with microRNA(miR)-146b, which is involved in premature ovarian failure (POF). This study was therefore carried out to explore its role in POF. In this study, 60 patients with POF and 60 controls were enrolled. The expression of BBOX1-AS1 and miR-146b were analyzed by RT-qPCRs. The direct interaction between miR-146b and the wild type BBOX1-AS1 (BBOX1-AS1-WT) or mutant BBOX1-AS1 (BBOX1-AS1-mut) was explored with RNA-RNA pulldown assay. Subcellular location of BBOX1-AS1 in COV434 granulosa cells was detected by subcellular fractionation. The role of BBOX1-AS1 and miR-146b in the apoptosis of COV434 cells was evaluated by cell apoptosis assay. Overexpression assay was applied to explore the relationship between BBOX1-AS1 and miR-146b. We found that the expression levels of BBOX1-AS1 were increased, while the expression levels of miR-146b were decreased in POF patients. BBOX1-AS1-WT, but not BBOX1-AS1-mut, directly interacted with miR-146b. BBOX1-AS1 was detected in both nucleus and cytoplasm, while they did not affect the expression of each other. BBOX1-AS1 suppressed the role of miR-146b in cell apoptosis. Therefore, BBOX1-AS1 may increase the apoptosis of granulosa cells in POF by sponging miR-146b.

## Graphical abstract

**Figure:**
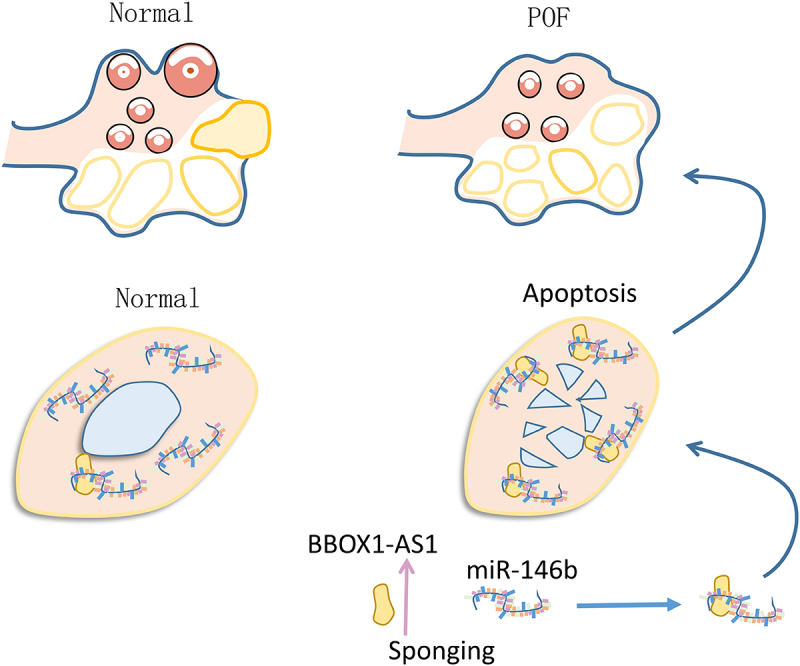

## Introduction

Premature ovarian failure (POF), commonly known as primary ovarian insufficiency, is a female-specific clinical disorder characterized by the development of hypoestrogenism, elevated gonadotrophins and occurrence of amenorrhea in women younger than 40 years old [1,2]. POF can be causes by many factors, such as metabolic disorders, toxins, autoimmune diseases, genetic factor and the application of radiation therapy and/or chemotherapy [3–5]. In effect, about 1 out of 100 women will be affected by POF by the age of 40 [6,7]. At present, POF is usually treated with hormone replacement therapy (HRT) [8]. However, HRT may cause a series of side effects, such as vaginal bleeding and depression [8]. Therefore, HRT is not appropriate for all patients. Currently, there is still no cure for POF, and all available approaches are developed to control the symptoms [9]. Therefore, novel therapies are of great importance for the treatment of POF.

It has been revealed that the development of POF requires the involvement of multiple genes, which form a complex gene regulatory network to affect disease progression [10]. Theoretically, certain genes with pivotal functions can be targeted to treat POF. For instance, reactivation of the Rictor/mTORC2 pathway, which is inactivated in POF, contributes to the recovery of POF [11]. However, more targets are needed to improve molecular targeted therapy for POF. Although several approaches have been tested in animal models to treat POF, clinical application is still difficult [12–15]. Long non-coding RNAs (lncRNAs) have no capacity in coding protein, but they participate in human diseases by regulating protein accumulation indirectly [16]. Therefore, lncRNAs may serve as potential targets to treat POF. LncRNA BBOX1 antisense RNA 1 (BBOX1-AS1) was reported to participate in ovarian cancer [17], while its role in other ovarian disorders is unclear. We speculated that BBOX1-AS1 could interact with microRNA (miR)-146b, which is involved in POF [18,19]. MiR-146b is known to downregulate CYP19A1 to increase the apoptosis of porcine ovarian granulosa cells [18]. In mouse model, miR-146b inactivates γH2A.X phosphorylation and the Dab2ip/Ask1/p38-Mapk pathway to attenuate POF [19]. Therefore, BBOX1-AS1 may participate in POF through the interaction with miR-146b. We then explored the role of BBOX1-AS1 in POF, with a focus on its interaction with miR-146b.

## Materials and methods

### Participants and specimens

POF patients (n = 60, 27.9 ± 6.6 years old) who received IVF/ICSI were enrolled in this study at General Hospital of Northern Theater Command after Ethics approval was obtained from the Ethics Committee of this hospital. POF was diagnosed by the following criteria: follicle stimulating hormone >30 IU/l in two measurements with an interval of 4 weeks. Patients who had autoimmune disease or other ovarian diseases were excluded from this study. The control group included 60 controls who received IVF/ICSI operation due to tubal factor infertility (n = 38) or male factor infertility (n = 22). Follicular fluid collection was performed during the IVF/ICSI operation. All participants signed the informed consent.

### Cell and cell culture

Functional *in vitro* assays were performed using COV434 granulosa cell line (Sigma-Aldrich). Mycoplasma contamination was checked, and only Mycoplasma-free cells were used. Following the instructions from ATCC, cells were cultivated in RPMI 1640 medium (10% FBS) supplemented with 100 U/mL and 100 μg/mL penicillin and streptomycin, respectively. Cells were cultivated in a cell culture incubator with humidity, CO_2_ and temperature set to 95%, 5% and 37 C, respectively.

### Cell transfection

Overexpression assays were performed by transfecting 10^8^ cells with the expression vector of BBOX1-AS1 (pcDNA3.1) and/or miR-146b mimic using Lipofectamine 3000 (Life Technologies). Both vector and miRNAs were prepared by GenePharma (Shanghai). Cytotoxicity was reduced by washing cells with fresh medium after incubation with transfection mixture (Lipofectamine 3000 + miRNA and/or vector) for 6 h. Overexpression was confirmed at 48 h post-transfection.

### RNA preparation

Isolation of total RNAs was carried out using the easy-BLUE™ Total RNA Extraction Kit (Labotaq). PureLink™ miRNA Isolation Kit (Thermo Fisher Scientific) was used for the isolation of miRNA. Briefly, cell lysis buffer was used to achieve cell lysis on ice for 30 min, and centrifugation was then performed at 12,000 g for 10 min to collect the supernatant, which was transferred to column for RNA binding. The columns were centrifuged at 12,000 g for 10 min, followed by washing with the washing buffer. Elution of RNA was performed by adding 40 ul RNase-free water followed by centrifugation at 12,000 g for 10 min.

### RT-qPCR

RNA integrity and concentration were both analyzed using 2100 Bioanalyzer. In all cases a RIN value higher than 8.5 and RNA concentration higher than 2,000 ng/ul were reached. Then 6, 000 ng RNA sample was used as the template to perform reverse transcriptions to prepare cDNA samples using the SSRT-IV kit (Thermo Fisher Scientific). Next, cDNA samples were used as the template to perform qPCR to measure the relative expression levels of BBOX1-AS1 and miR-146b with 18S rRNA as the internal control. Ct values were processed using the 2^−ΔΔCt^ method [20] to calculate relative expression levels of BBOX1-AS1 and miR-146b.

### Subcellular fractionation assay

The preparation of both cytoplasm and nucleus samples of cells was performed using the PARIS™ Kit (Invitrogen). Cells were washed with PBS and then counted. The washed cells (10^8^) were then subjected to cell lysis by incubating cells with cell lysis buffer for 10 min on ice. The cytoplasm was separated by centrifuging cell lysate at 12,000 g for 10 min, followed by collection of the supernatant. Nucleus lysis was then performed by incubating cell pellets with nucleus lysis buffer on ice for 10 min. RNA isolation was then performed, followed by RT-PCR to detect the expression levels of BBOX1-AS1 with GAPDH as an cytoplasm marker.

### RNA pulldown assay

To explore the direct binding of miR-146b and BBOX1-AS1, *in vitro* transcriptions were performed using T7 RNA polymerase (Invitrogen) to prepare *in vitro* transcripts of the wild type BBOX1-AS1 (BBOX1-AS1-WT), mutant BBOX1-AS1 (BBOX1-AS1-mut) and NC. These transcripts were labeled with biotin, followed by transfection into cells. Cells were collected at 48 h post-transfection to perform cell lysis, followed by using Streptavidin-Dyna beads (Invitrogen) to pulldown RNA samples. The beads were then used to perform RNA isolation, followed by RT-qPCR to determine the expression of miR-146b.

### Dual luciferase reporter assay

The dual luciferase reporter assay was performed as previously described [21]. BBOX1-AS1-WT and BBOX1-AS1-mut were cloned into pGL3 vector to prepare luciferase vector. Then two luciferase vector or empty control vector was co-transfected with miR-146b mimic into COV434 cells. Luciferase activity was determined at 48 h later.

### Cell apoptosis assay

Cells with transfections were collected at logarithmic phase and washed with PBS. After counting, cells were transferred to a 6-well plate with 1 × 10^6^ cells in 1 ml medium per well. After that, each well was added with 200 μl of Annexin V-FITC, followed by incubation in dark for 15 min. After that, 200 μl of propidium iodide was added. Then flow cytometry was performed to analyze cell apoptosis using BD FACSCelesta™ Flow Cytometer.

### Statistical methods

Comparisons of datasets and plotting of images were performed using the SPSS 19.0 software. Two groups were compared using Student’s t test. P < 0.05 was statistically significant.

## Results

### Differential expression of BOX1-AS1 and miR-146b in follicular fluid from both POF patients and the control

**Figure 1.**
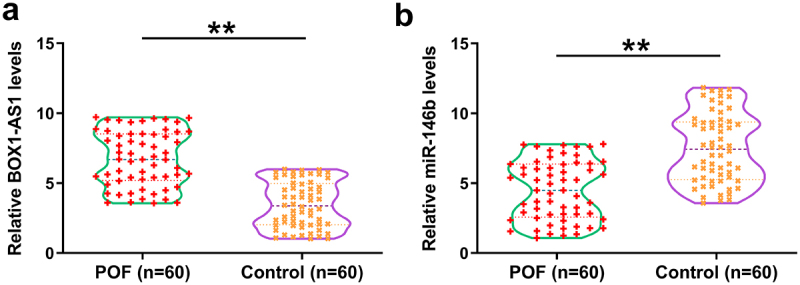
The expression of BOX1-AS1 and miR-146b in follicular fluid. Follicular fluid from 60 POF patients and 60 controls were collected, followed by RNA isolation and RT-qPCRs to analyze the expression of BBOX1-AS1 (a) and miR-146b (b). **, *p* < 0.01.

Gene expression analysis is critical for function analysis. Therefore, the differential expression of BBOX1-AS1 and miR-146b in follicular fluid from the 60 POF patients and the 60 controls were first analyzed. It showed that the expression levels of BBOX1-AS1 were significantly increased in POF patients (Figure 1(a), *p* < 0.01), and the expression levels of miR-146b were significantly decreased (Figure 1(b), *p* < 0.01) compared with that in the control. Therefore, altered expression of BBOX1-AS1 and miR-146b might be involved in the progression of POF.

### The binding of miR-146b to BBOX1-AS1

**Figure 2.**
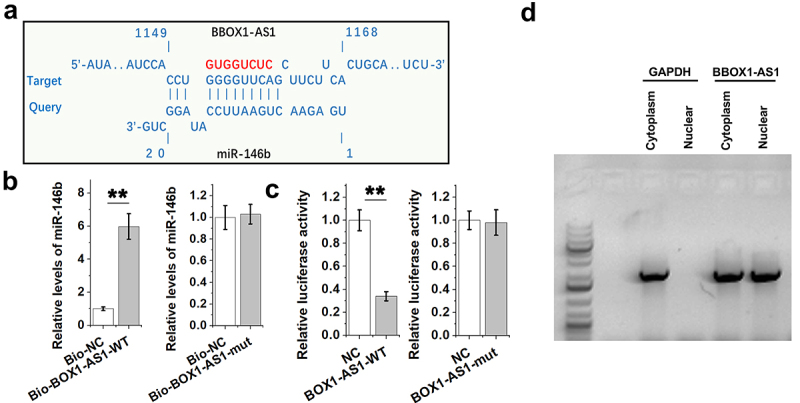
The binding of miR-146b to BBOX1-AS1. IntaRNA 2.0 online program was applied to explore the potential interaction between them (a). Mutant BBOX1-AS1 (BBOX1-AS1-mut) with disrupted binding sites of miR-146b was designed and indicated by red color. The direct interaction between miR-146b and the wild type BBOX1-AS1 (BBOX1-AS1-WT) or BBOX1-AS1-mut was explored with RNA-RNA pulldown assay (b). Dual luciferase report assay was also performed to further confirm the interaction between them (c). Subcellular location of BBOX1-AS1 in COV434 granulosa cells was analyzed by subcellular fractionation (d). **, *p* < 0.01.

Direct binding indicates interactions and could predict gene function. IntaRNA 2.0 online program was applied to explore the potential interaction between miR-146b and BBOX1-AS1. It was predicted that miR-146b might bind with BBOX1-AS1 (Figure 2(a)). Mutant BBOX1-AS1 (BBOX1-AS1-mut) with disrupted binding sites of miR-146b was designed and indicated by red color. And the direct interaction between miR-146b and BBOX1-AS1-WT or BBOX1-AS1-mut was explored with RNA-RNA pulldown assay. The results showed that Bio-BBOX1-AS1-WT, but not Bio-BBOX1-AS1-mut, showed increased levels of miR-146b compared to that in Bio-NC group (Figure 2(b), *p* < 0.01). Dual luciferase report assay was also performed to further confirm the interaction between them. It was observed that miR-146b only decreased the luciferase activity of BBOX1-AS1-WT, but not that of BBOX1-AS1-mut. Therefore, BBOX1-AS1-WT, but not BBOX1-AS1-mut, directly interacted with miR-146b. Subcellular location of BBOX1-AS1 in COV434 granulosa cells was analyzed by subcellular fractionation. Different from GAPDH (cytoplasm-specific), BBOX1-AS1 was detected in both nucleus and cytoplasm (Figure 2(d)). Therefore, BBOX1-AS1 may interact with miR-146b.

### Regulatory role of BBOX1-AS1 and miR-146b in regulating the expression of each other

**Figure 3.**
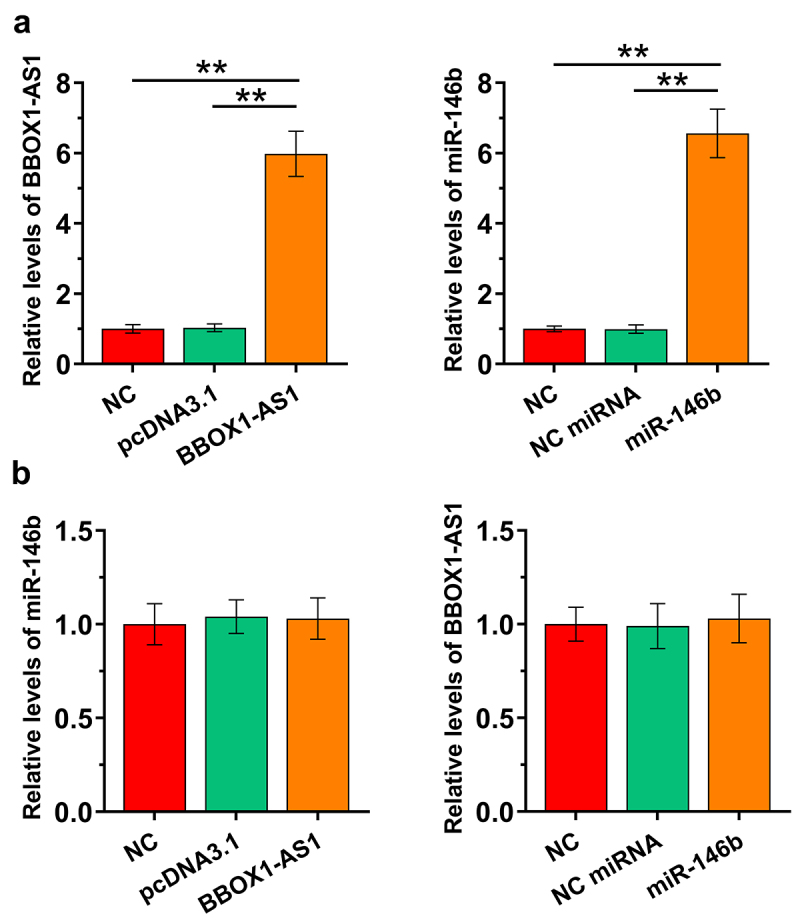
The effects of overexpression of BBOX1-AS1 and miR-146b on regulating the expression of each other. COV434 granulosa cells were overexpressed with BBOX1-AS1 or miR-146b to study the crosstalk between them. Overexpression was confirmed at 48 h after the initiation of transfection (a). After transfection, miR-146b expression in cells with BBOX1-AS1 overexpression (b) and BBOX1-AS1 expression in cells with miR-146b overexpression (c) were both analyzed with RT-qPCRs. **, *p* < 0.01.

Our data presented above suggested potential interaction between BBOX1-AS1 and miR-146b. COV434 granulosa cells were then overexpressed with BBOX1-AS1 or miR-146b to study the crosstalk between them. The overexpression was confirmed at 48 h post-transfection (Figure 3(a), *p* < 0.01). After transfection, the expression of miR-146b in cells with the overexpression of BBOX1-AS1 (Figure 3(b)), and the expression of BBOX1-AS1 in cells with the overexpression of miR-146b (Figure 3(c)) were both analyzed by RT-qPCRs. The results showed that they did not affect the expression of each other. These data suggested that BBOX1-AS1 was not a target of miR-146b.

### The role of BBOX1-AS1 and miR-146b in cell apoptosis

**Figure 4.**
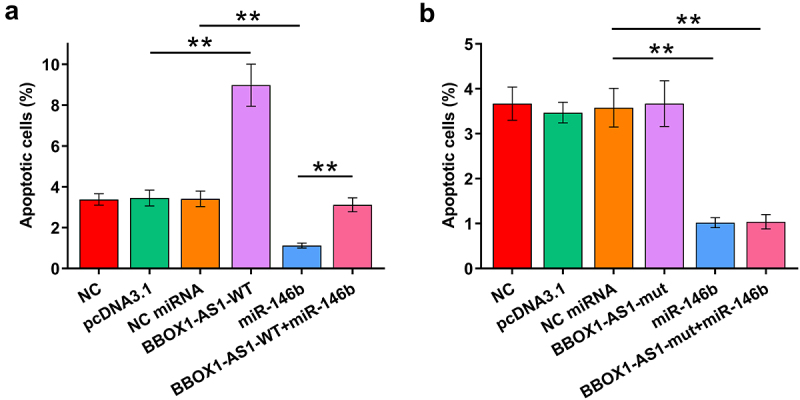
Analysis of the role of BBOX1-AS1 and miR-146b in cell apoptosis. The role of BBOX1-AS1-WT (a), BBOX1-AS1-mut (b) and miR-146b in the apoptosis of COV434 cells was analyzed by cell apoptosis assay. **, *p* < 0.01.

Cell apoptosis contributes to POF. The role of BBOX1-AS1-WT, BBOX1-AS1-mut, and miR-146b in the apoptosis of COV434 cells was therefore analyzed by cell apoptosis assay. Cells were transfected with BBOX1-AS1 (-WT or -mut) vector and co-transfection with miR-146b was also performed. It showed that BBOX1-AS1-WT increased cell apoptosis, while miR-146b decreased cell apoptosis. Moreover, BBOX1-AS1-WT suppressed the role of miR-146b in regulating the cell apoptosis (Figure 4(a), *p* < 0.01). In contrast, BBOX1-AS1-mut did not affect cell apoptosis and showed no effects on the regulation of miR-146b in cell apoptosis (Figure 4(b), *p* < 0.01). Therefore, BBOX1-AS1-WT may promote cell apoptosis in POF by sponging miR-146b.

## Discussion

BBOX1-AS1 has been reported to participate in multiple human diseases, while its role in POF is unclear. The present study explored the involvement of BBOX1-AS1 in POF and assessed its interaction with miR-146b. We observed altered expression of BBOX1-AS1 and miR-146b in POF. Our results also demonstrated that BBOX1-AS1 could sponge miR-146b in POF to promote cell apoptosis.

BBOX1-AS1 participates in multiple human cancers. For instance, BBOX1-AS1 is highly expressed in ovarian cancer, where BBOX1-AS1 augments PODXL by sequestering miR-361-3p to promote cancer progression [17]. To date, the participation of BBOX1-AS1 in other human diseases is unclear. We showed that BBOX1-AS1 is highly expressed in POF patients, suggesting its potential involvement in POF. Granulosa cells regulate the development of female gamete, and increased apoptosis of these cells contributes to POF. In effect, inhibition of the apoptosis of granulosa cells can improve POF in animal models [22]. In the present study we showed that BBOX1-AS1 could increase the apoptosis of granulosa cells. Therefore, BBOX1-AS1 may promote POF by promoting granulosa cell apoptosis.

Previous studies have reported opposite roles of miR-146b in POF. One study reported that upregulation of miR-146b promoted the apoptosis of granulosa cells by downregulating CYP19A1 [18]. In another study, it was reported that the expression of miR-146b could mitigate the aging of granulosa cells by inactivating the Dab2ip/Ask1/p38‐Mapk pathway [19]. Our study reported the downregulation of miR-146b in POF and observed the inhibitory effects of miR-146b on the apoptosis of granulosa cells. Therefore, miR-146b plays a protective role in POF.

The regulation of miR-146b in POF is unclear. We showed that miR-146b could bind to BBOX1-AS1, while they have no role in regulating the expression of each other. However, BBOX1-AS1-WT suppressed the role of miR-146b in cell apoptosis, while BBOX1-AS1-mut with disrupted binding sites of miR-146b showed no effect on cell apoptosis. Therefore, the function of BBOX1-AS1 in POF is likely mediated by miR-146b. Because BBOX1-AS1 could be detected in both cytoplasm and nucleus, and mature miRNAs are mainly enriched in cytoplasm, we speculated that BBOX1-AS1 in cytoplasm may sponge miR-146b to suppress it role in cell apoptosis. Although previous studies have extensively investigated the pathogenesis of POF and molecular players involved in this disease [23,24], more studies are still needed to identify safe and effective molecular targets to treat POF.

## Conclusion

BBOX1-AS1 is highly upregulated in POF and it may promote POF by increasing cell apoptosis. The role of BBOX1-AS1 in POF is likely achieved by serving as an ceRNA of miR-146b.

## Data Availability

The analyzed data sets generated during the study are available from the corresponding author on reasonable request.
